# Biomedical engineering meets acupuncture - development of a miniaturized 48-channel skin impedance measurement system for needle and laser acupuncture

**DOI:** 10.1186/1475-925X-9-78

**Published:** 2010-11-23

**Authors:** Gerhard Litscher, Lu Wang

**Affiliations:** 1 Research Unit of Biomedical Engineering in Anesthesia and Intensive Care Medicine and TCM Research Center Graz, Medical University of Graz, Auenbruggerplatz 29, 8036 Graz, Austria

## Abstract

**Background:**

Due to controversially discussed results in scientific literature concerning changes of electrical skin impedance before and during acupuncture a new measurement system has been developed.

**Methods:**

The prototype measures and analyzes the electrical skin impedance computer-based and simultaneously in 48 channels within a 2.5×3.5 cm matrix. Preliminary measurements in one person were performed using metal needle and violet laser (405 nm) acupuncture at the acupoint Kongzui (LU6). The new system is an improvement on devices previously developed by other researchers for this purpose.

**Results:**

Skin impedance in the immediate surroundings of the acupoint was lowered reproducibly following needle stimulation and also violet laser stimulation.

**Conclusions:**

A new instrumentation for skin impedance measurements is presented. The following hypotheses suggested by our results will have to be tested in further studies: Needle acupuncture causes significant, specific local changes of electrical skin impedance parameters. Optical stimulation (violet laser) at an acupoint causes direct electrical biosignal changes.

## Background

The autonomic nervous system plays a key role in basic acupuncture research [1]. There are several studies evaluating the electrical properties of acupuncture points and meridians [2]. For the first time we are able to investigate possible acupuncture-specific changes in the activity of the autonomic nervous system using a newly developed prototype which measures and analyzes the electric skin impedance computer-based and simultaneously in 48 spots within a 2.5×3.5 cm matrix.

A review of scientific literature on the topic yields 320 publications dealing with electrical skin impedance measurements. Considering only those using methods of evidence-based medicine, this number is reduced to 18 relevant studies [2]. These can be divided into acupuncture studies (n = 9) and so-called 'meridian studies' (n = 9). Five of the acupuncture studies found a lowered skin impedance in the area of an acupuncture point, four studies yielded contrary results.

Because of the controversially discussed results of existing studies, a new multi-channel skin impedance measurement system was developed at the TCM (Traditional Chinese Medicine) Research Center at the Medical University of Graz. This system was designed to supply objective data for the first time, taking into consideration the previously existing technical limitations. The present manuscript introduces the new measurement system and contains first problem-oriented, forward-looking results on needle and laser acupuncture.

Relevant previous work by other authors has to be pointed out: The publication by Becker et al. [3] laid the original foundation for our miniaturized 48-channel-system. More recently, the research group of Wiegele et al. [4] designed and the research group of Kramer et al. [5] tested a similar device. The device designed by Wiegele et al., however, did not have a spatial resolution as precise as the one presented in this paper, but is an important forerunner of this system.

Main reasons for the development of this system were to eventually test the following three hypotheses:

(i) Does the skin resistance show differences between acupoints and the surrounding area already in resting state?

(ii) Does needle acupuncture and needle stimulation result in any significant local changes of electrical skin resistance?

(iii) Does violet laser acupuncture alter skin resistance?

## Methods

**Figure 1:**
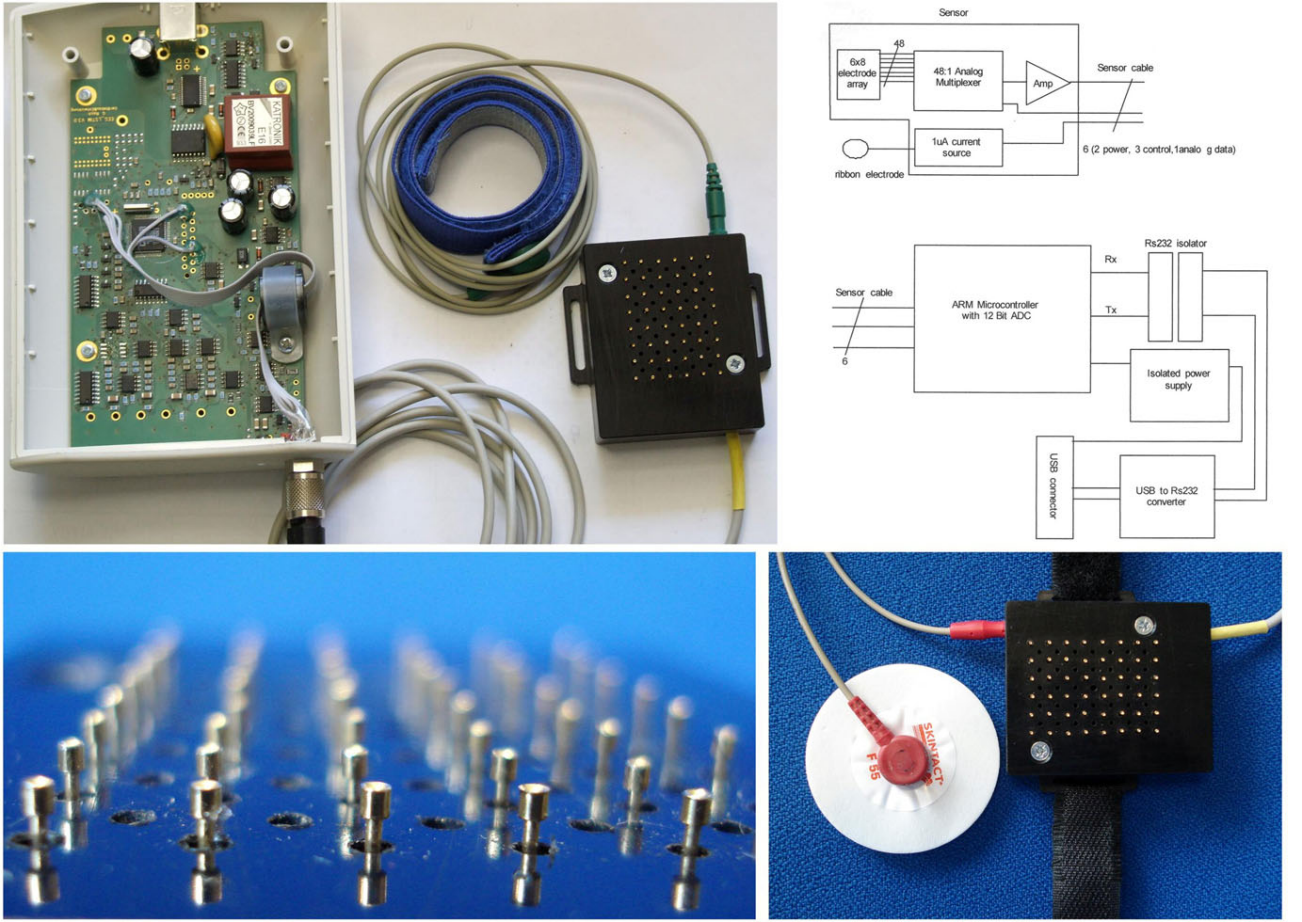
**Technical details of the newly developed acupuncture impedance measurement system**. From left to right and top to bottom: Printed circuit board assembly, measurement system with grounding electrode for the wrist including a hook-and-loop fastener, sensor unit; circuit diagram; 48 spring load mounted skin electrode contact cylinders; ECG grounding electrode compared in size to the sensor part of the measurement system.

The new prototype DC (direct current) system was developed to measure skin impedance at the location of an acupoint over seconds to hours. The measurement circuit is time multiplexed across 48 channels. The electrodes have a diameter of 0.9 mm and the material is gold-plated beryllium copper. It is not possible to measure the pressure under each probe during monitoring skin resistance; however, the force of the 48 electrodes is between 0.5 - 1 N. A part of the technical specifications can be seen in Figure 1 along with the new construction of the system, realized in May 2010. A calibration against a standard is not possible because there is no standard device available at the moment.

**Figure 2:**
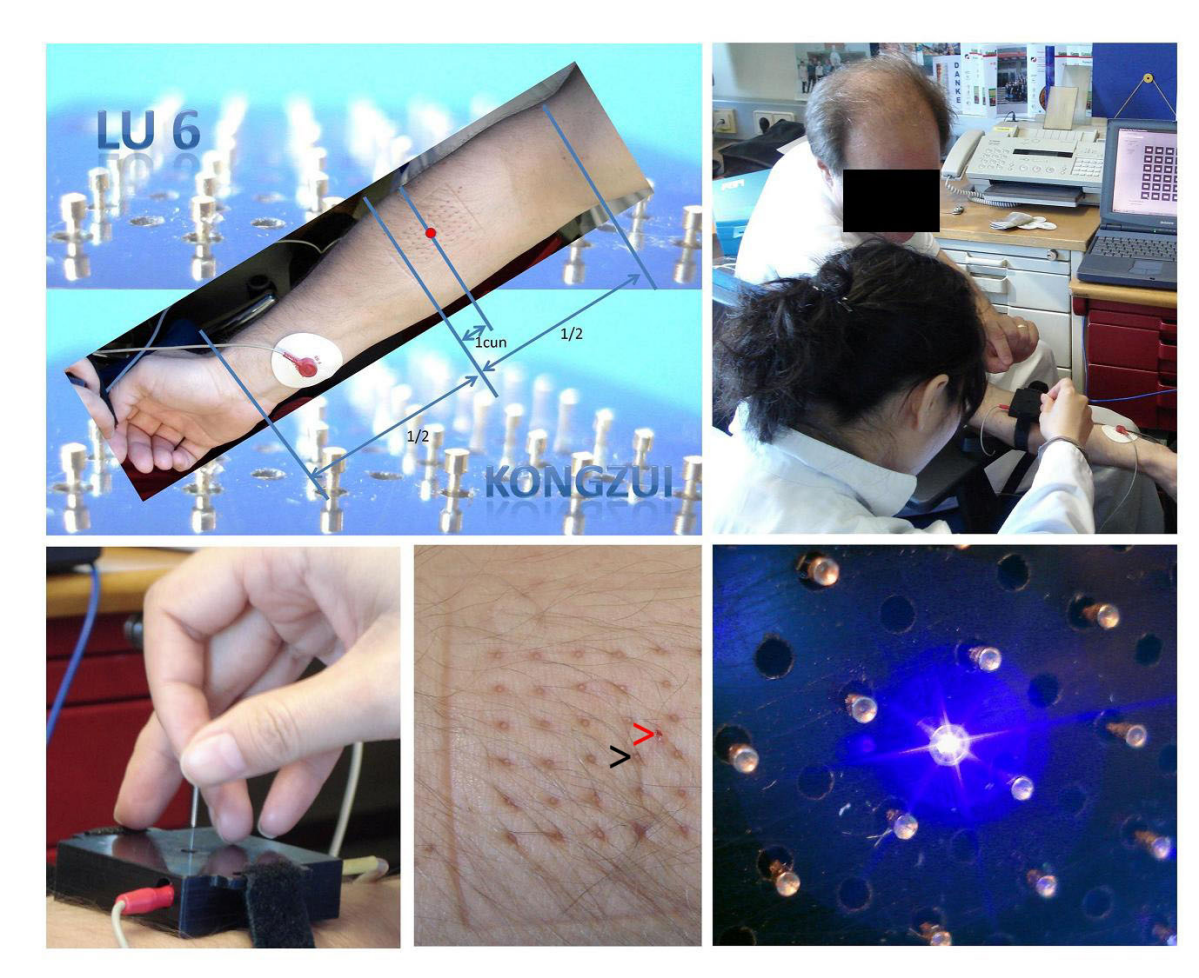
**Acupuncture point and practical details of the new impedance measurement system**. From left to right and top to bottom: Localization of the acupuncture point Lu 6 (Kongzui); documentation of the first measurement performed at the TCM Research Center Graz (May 7, 2010); application and manual stimulation of a metal needle via the system's guiding rail provided for this purpose; puncture site of the needle (red arrow) compared to the indentations of the miniaturized measurement cylinders (e.g. black arrow); violet laser (405 nm) stimulation between the registration sites (distance 2.5 mm).

First preliminary pilot measurements were performed in one subject to test the validity of the new method. The investigations were carried out in constant environmental conditions (room temperature 23°C). The skin was cleaned with alcohol in the area where registration was to take place, and all metallic and/or conductive items were removed from the body. In a sitting position, registrations were carried out using an electrical current of 1.46 μA. The resting interval before starting the measurement was 10 min. Apart from baseline registrations, acupuncture stimuli (metal needle (0.3 × 50 mm, silicone-coated steel (Seirin Corp., Shizuoka, Japan); penetration depth 1 cun, needling perpendicular) or violet laser (405 nm, 110 mW, 500 μm, [6])) were applied at acupoint Lu6 (Kongzui, comp. Figure 2) and their effects were quantified in the pilot measurements using the new system. The acupoint was not chosen for therapeutical, but for practical reasons (application of the system). The location of the acupoint was located by an experienced acupuncturist. Point location was based on cun measurement (see Figure 2) on the one side, on the other side on anatomical basis (on the radial side of the palmar surface of the forearm, along the lateral border of the lower end of m. pronator teves, the medial borders of m. extensor carpi radialis longus and brevis). For each condition (metal needle/violet laser), two measurements have been performed; typical examples are shown in the results section, but of course no statistical analysis has been performed. The time between the single measurements was at least one day. Deqi was elicited during both metal needle and violet laser stimulation. The measurement procedure can also be seen in Figure 2.

## Results and Discussion

**Figure 3:**
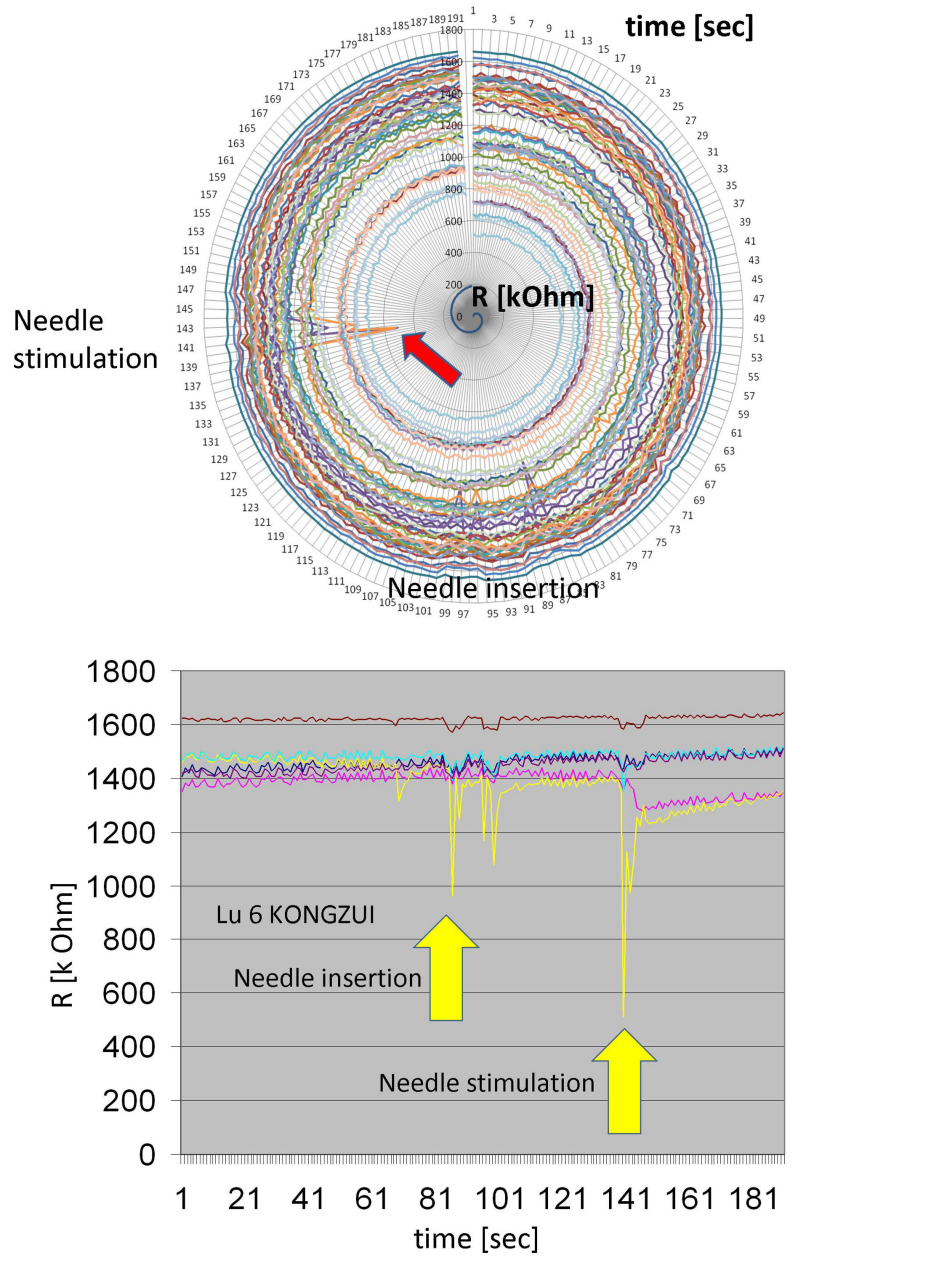
**Analyses of the 48 channels of the impedance system during needle insertion and needle stimulation**. Top: Circular chart - needle stimulation (time in sec clockwise and resistance R in kOhm from center outwards). Bottom: Changes in electrodermal impedance during manual needle stimulation (note the lowered impedance in some, but not all channels). Six channels surrounding the acupoint were randomly chosen. In this measurement, the most marked change in impedance during insertion and stimulation of the metal needle can be found in one electrode (yellow line) next (2.5 mm) to the acupoint.

**Figure 4:**
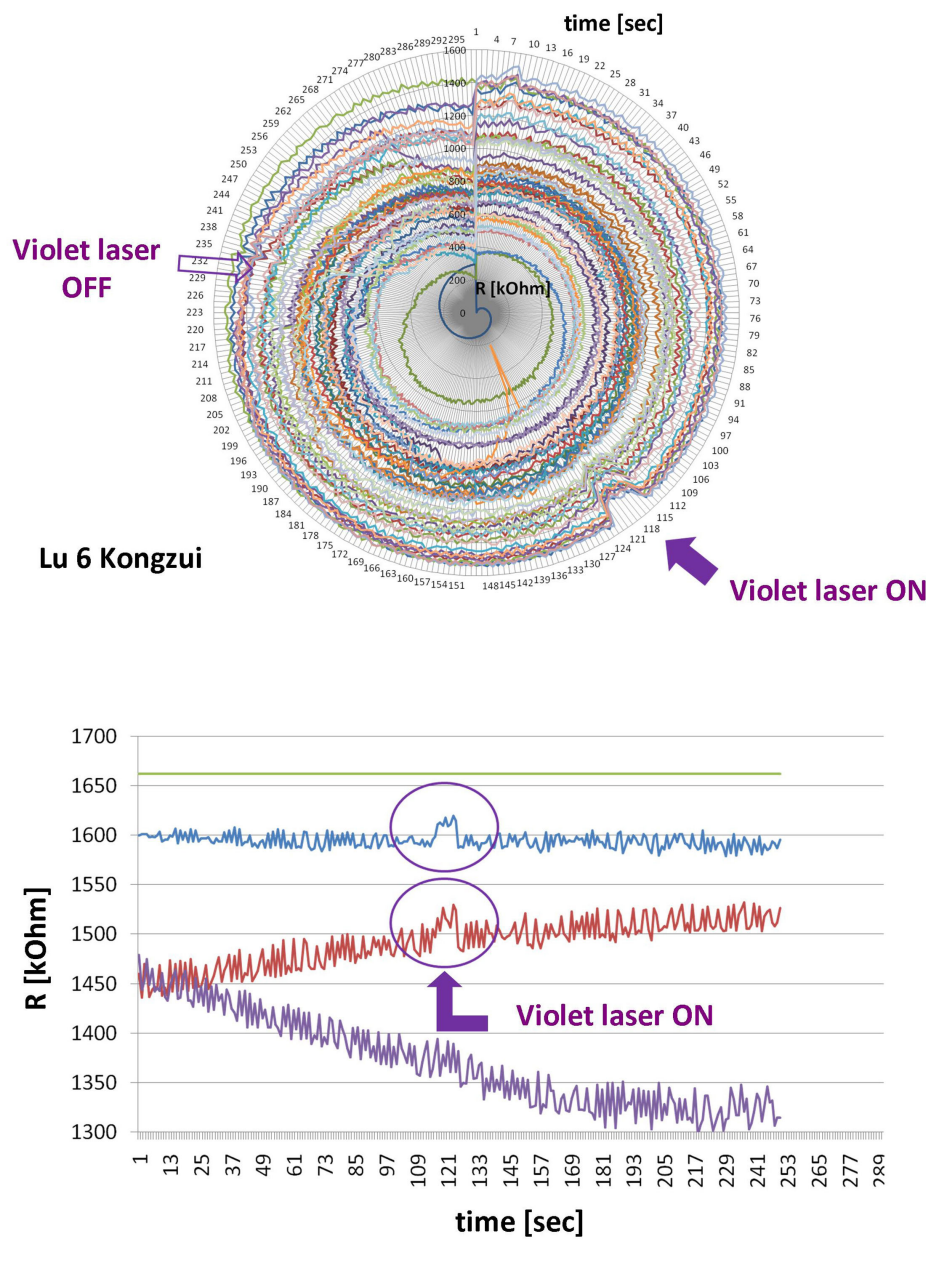
**First analysis of changes of impedance during contact-free violet laser stimulation**. Top: Circular chart - violet laser acupuncture, details see Figure 3. Bottom: Changes in electrodermal impedance immediately following the onset of violet laser stimulation in specific registration sites only. Three channels surrounding the acupoint were randomly chosen. In this measurement, the 'on-effect' of the violet laser is clearly demonstrated.

All recordings were reliable at all 48 skin sites. The first results showed very interesting changes in skin impedance in several registration channels. For example, skin impedance in the immediate surroundings of the acupoint was lowered reproducibly following manual needle stimulation (Figure 3). In case of violet laser stimulation these phenomena could also be observed in an evidence-based manner (Figure 4).

As mentioned in the background section, there are some already existing important systems. Already in 1976, Becker et al. [3] have performed very interesting measurements with a system based on the same idea (multi-channel recordings). However, the data are not directly comparable because they used different materials, different inter-electrode distances and also a different recording procedure. They first investigated the skin with a meridian scanning probe (1-channel system) and then with the 36-channel system. One of the main disadvantages of this previously developed system was that investigations can only be performed in the resting period, but not during acupuncture stimulation. With our system, it is possible for the first time to continuously and simultaneously monitor a region of interest (around the acupoint) even during the insertion of a metal needle or the activation/deactivation of a laser for acupuncture.

The previously existing limitations of electrodermal impedance measurements were the measurement area (point selectors with a tip only, representing a hand-held 1-channel system) and related problems (pressure, angle) and also too few registration sites (in most cases only punctual measurements, no multi-channel systems)[7,8]. In contrast to our electrode configuration (electrode diameter 0.9 mm), the electrodes of the system of Colbert et al. [8] have a diameter of 4 mm and are fixed separately at the body surface with an elastic adhesive or cloth wrist band. "Confounding factors, such as skin moisture, electrode pressure, stratum corneum thickness, electrode polarization and other factors, have led many to assert that the reportedly distinct electrical characteristics are attributable to external factors and/or artifacts and not to the acupuncture point or meridian" [2]. The newly developed system allows for the first time simultaneous and continuous, acupuncture-relevant multi-channel measurements using appropriate analysis methods. As we have only performed measurements in one subject, further measurements in a great number of healthy volunteers are absolutely necessary.

## Conclusions

The present paper is principally a demonstration of a new instrumentation. In addition, the preliminary results suggest hypotheses listed below (i - iii) which will be tested by further studies:

*(i) Skin resistance within a distance of 2.5×3 cm from an acupoint shows distinct differences already in resting state*. *These variations and their connections with different acupoints have to be clarified in extensive studies;*

(ii) Needle insertion and manual needle stimulation cause significant, specific and local changes of electrical skin resistance parameters;

*(iii) It is noteworthy that also acupuncture stimulation using a violet ('blue') laser (405 nm; 110 mW, 500 μm) can alter skin resistance significantly, specifically and locally*.

The latter result (iii) could be the first proof in acupuncture research that **optical **stimulation (violet laser) at an acupoint causes a direct **electrical **biosignal change at a distance of < 3 mm. This fact alone might yield answers to important core questions in acupuncture (meridian and/or placebo research), be it for further basic or clinical acupuncture research.

## Competing interests

The authors declare that they have no competing interests.

## Authors' contributions

GL and LW conceived and designed the system and the preliminary study. LW was responsible for all issues concerning Traditional Chinese Medicine, and GL was responsible for biomedical engineering issues. Both authors read and approved the final manuscript.
